# Elucidation of Architectural and Compositional Factors Associated With Inter‐Individual Variability in Passive Shear Modulus of the Human Vastus Lateralis in Young Healthy Males

**DOI:** 10.1111/sms.70348

**Published:** 2026-07-10

**Authors:** Raki Kawama, Yuki Okuda, Satoshi Mikata, Kippei Sato, Katsuki Takahashi, Freek Van de Casteele, Eline Lievens, Wim Derave, Taku Wakahara

**Affiliations:** ^1^ Faculty of Sport Sciences Waseda University Saitama Japan; ^2^ Organization for Research Innovation Doshisha University Kyoto Japan; ^3^ Faculty of Health and Sports Science Doshisha University Kyoto Japan; ^4^ Graduate School of Health and Sports Science Doshisha University Kyoto Japan; ^5^ Department of Movement and Sports Sciences Ghent University Ghent Belgium; ^6^ Human Performance Laboratory Waseda University Saitama Japan

**Keywords:** fascicle length, intramuscular fat content, muscle fiber type composition, muscle mechanical properties, pennation angle

## Abstract

Human skeletal muscle exhibits substantial inter‐individual heterogeneity in passive mechanical properties; however, the mechanistic basis underlying this variability remains poorly understood. We aimed to comprehensively examine how compositional and architectural factors are associated with individual variability in passive shear modulus of the human vastus lateralis (VL). In 46 healthy young males, the shear modulus of VL was measured from 0° to 110° of knee flexion in 10° increments using ultrasound shear wave elastography to determine the slack angle (angle at which the shear modulus first began to increase), shear modulus in the slack region (values before the slack angle), and Δshear modulus in the lengthened position (+35° from the slack region). Muscle carnosine concentration (a proxy for muscle fiber type composition), intramuscular fat fraction, 3D muscle architecture (fascicle length and pennation angle), and knee extensor moment arm were quantified using a magnetic resonance scanner. The inter‐individual variability was greater for the Δshear modulus in the lengthened position (54.5% [coefficient of variation]) than for the shear modulus in the slack region (16.4%). The shear modulus in the slack region was not associated with any of the measured variables, whereas Δshear modulus in the lengthened position was only associated with pennation angle (corrected *p* = 0.004, *r* = −0.488), explaining approximately 20% of the variance. These findings indicate that pennation angle is associated when the muscle is lengthened for a given joint angle change; yet even this factor explains only a limited portion of the inter‐individual variability in VL passive shear modulus.

## 1 Introduction

Human skeletal muscle exhibits substantial inter‐individual heterogeneity in passive mechanical properties, often presented as shear modulus (up to 80% of the coefficient of variation [CV]) [1]. Such inter‐individual variability has been shown to be positively associated with differences in physical performance, such as explosive force production capacity [2], as well as susceptibility to muscle tissue failure [3]. Despite the clear physiological and clinical relevance, we still lack a mechanistic understanding of the factors responsible for such inter‐individual variability.

Potential factors contributing to the inter‐individual variability in passive muscle shear modulus are the compositional variables (e.g., fiber type composition and intramuscular fat content) of human skeletal muscle. Skeletal muscle is composed of multiple tissue components with different elastic properties, including contractile fibers (fast‐twitch and slow‐twitch fibers) and adipose tissue. Under passive conditions, slow‐twitch fibers were shown to be stiffer than fast‐twitch fibers [4], possibly due to their more robust cytoskeletal structures, such as larger Z‐discs, titin, nebulin, and M‐band proteins, and abundant collagenous connective tissues [4, 5]. Additionally, muscle tissue was shown to be stiffer than adipose tissue [6]. Thus, the relative proportions of these components—fast‐ versus slow‐twitch fibers, and adipose versus muscle tissues—within a given muscle may contribute to inter‐individual variability in passive muscle shear modulus.

Other factors that may influence the inter‐individual variability in passive muscle shear modulus are the muscle architectural variables, such as moment arm (MA), pennation angle, and fascicle length. Theoretically, muscles with a shorter MA (defined as the perpendicular distance from the joint center of rotation to the muscle's line of action) undergo smaller overall muscle‐tendon unit elongation for a given change in joint angle than muscles with a longer MA [7]. Additionally, in pennate muscles, fascicles attach to the intramuscular part of tendon (i.e., aponeurosis) with an angle relative to the long axis of the muscle belly in the sagittal (perpendicular to the aponeurosis) and coronal (parallel to the aponeurosis) planes [8, 9]. This 3D fascicle arrangement allows them to rotate in both planes during muscle belly lengthening. Thus, the amount of fascicle elongation relative to muscle belly length change would be small for a given joint angle change—a phenomenon known as the “gearing effect” [9]. Moreover, each fascicle length is determined as the product of the number of sarcomeres in series and length per sarcomere. A greater number of sarcomeres arranged in series would result in smaller elongation of each sarcomere for a given fascicle elongation [10]. Consequently, based on the above theoretical framework, the fascicle length relative to MA and pennation angle may also influence the inter‐individual variability in the change in passive shear modulus, especially when the muscle belly is lengthened for a given joint angle change.

To date, previous studies reported considerable individual variability in factors such as intramuscular fat content [11], fiber typology [12], fascicle length [13], pennation angle [14], and MA [15]. However, given the complex interplay of these components within skeletal muscle, it remains unclear how individual compositional and architectural factors influence the inter‐individual variability in passive muscle shear modulus, except for a study reporting the negative relationship between intramuscular fat content and change in passive muscle shear modulus [11]. Here, we comprehensively examined whether compositional (intramuscular fat content and fiber typology) and architectural (three‐dimensional fascicle architecture and MA) factors of human skeletal muscle are associated with the inter‐individual variability in passive shear modulus of the vastus lateralis (VL) muscle, using a combination of advanced imaging techniques: ultrasound shear wave elastography, proton magnetic resonance spectroscopy (^1^H‐MRS), DIXON magnetic resonance (MR) imaging, and diffusion tensor imaging (DTI) and tractography. We hypothesized that higher intramuscular fat content and a smaller estimated proportion of slow‐twitch fibers (i.e., higher carnosine concentration) would be associated with lower passive muscle shear modulus in the non‐lengthened (slack) region, whereas, during muscle lengthening for a given change in joint angle, the higher ratio of fascicle length to MA and a larger pennation angle would be associated with a smaller increase in passive muscle shear modulus.

## 2 Methods

### 2.1 Participants & Experimental Design

A prior power analysis was performed to determine the ideal sample size required for significant correlation between architectural and/or compositional variables and muscle shear modulus with a power of 80%, an α error of 0.05, and an effect size (*r*) of 0.40 for a two‐tailed correlation test, using G*Power 3.1 software (Heinrich Heine University, Dusseldorf, Germany). The effect size of 0.40 was selected based on a previous study reporting significant correlation between intramuscular fat content and change in muscle shear wave velocity between relatively short and long muscle lengths (*r* = 0.47) [11]. The minimal sample size was calculated to be 44, and thus 46 healthy young males (age, 22.8 ± 4.2 years; body height, 172.2 ± 4.8 cm; body mass, 64.3 ± 9.0 kg) were recruited for this study to account for the risk of participant attrition. The participants had not engaged in a regular routine of resistance training for at least 1 year prior to the study and had no history of injuries to the quadriceps femoris muscles. This study did not include individuals who were vegetarian or vegan, or had taken β‐alanine or any other supplements within the 3 months prior to or during the study because such dietary habits could influence muscle carnosine concentrations [16]. A written informed consent form was obtained from all participants after the purpose, procedures, and possible risks and burdens of the present study were carefully explained to them in writing and verbally. This study was approved by the Ethics Committee of the authors' institution (No. 24060) and was conducted based on the principles of the Declaration of Helsinki.

In the present study, muscle shear modulus and other variables (compositional and architectural factors) were measured across two experimental days at our University. On the first day, the shear modulus of the right VL was measured at rest using ultrasound shear wave elastography to determine the slack angle (i.e., the joint angle at which the shear modulus first began to increase), the shear modulus in the slack region (i.e., shear modulus values before the slack angle), and the Δshear modulus in the lengthened position (+35° from the slack region). On the second day, muscle carnosine concentration (a proxy for fiber type composition) and intramuscular fat fraction were quantified using proton ^1^H‐MRS and two‐point DIXON MR imaging, respectively. Additionally, three‐dimensional muscle architecture (fascicle length and pennation angle) and the knee extensor MA were quantified using DTI and T1‐weighted MR images, respectively. This study focused on VL because this muscle is a pennate muscle with inter‐individual variability in passive shear modulus (CV of 28 to 44%) [17] and showed a measurement validation of the aforementioned factors using an MR scanner [12, 18, 19]. Several mechanisms have been proposed that could theoretically influence passive muscle mechanical properties, including cross‐bridge dynamics between myosin and actin [20], titin [21], and intramuscular connective tissues such as the endomysium and perimysium [22]. However, direct assessment of these microscopic determinants was beyond the scope of the present study because the required measurements are invasive and/or not feasible with the equipment available at our institution (see Future directions). Thus, as a first step toward identifying contributors to inter‐individual variability in passive shear modulus, we focused on the aforementioned compositional and architectural factors.

### 2.2 Muscle Shear Modulus

The participants were seated on the bench of a dynamometer (Biodex System4, Biodex Medical Systems, USA) with the right knee fully extended (0° flexion). The hip joint was slightly flexed at 30°, because excessive lengthening of the rectus femoris may change the shear modulus of the adjacent muscles in the fully extended hip position, possibly due to epimuscular myofascial force transmission. The rotation axis of the right knee joint was carefully aligned with that of the dynamometer, and the right lower leg was secured to the lever arm. The trunk and pelvis were securely fixed to the dynamometer bench using a non‐elastic belt.

To assess the shear modulus of VL, we used an ultrasound scanner (Aixplorer Ver. 8, Supersonic Imagine, France) in shear wave elastography mode (MSK preset, penetrate mode, persistence = High, smoothing level = 5, scale = 0–180 kPa), equipped with a linear array transducer (4–15 MHz; SuperLinear 15–4; Vermon, France). The transducer was placed at 50% of thigh length (the distance between the greater trochanter [0%] and the popliteal crease [100%]) and at 50% of VL width (the distance between its borders with the biceps femoris long head and the rectus femoris). In these positions, the fascicle orientation of VL was inspected by B‐mode images and marked on the skin with a permanent marker to ensure consistent placement. The shear modulus measurements were performed from 0° to 110° of knee flexion at 10° increments. The shear wave elastography images were obtained five times (within 2 min) at 0° of knee flexion (for the calculation of the muscle slack angle described later) and two times (within 1 min) at the other knee joint angles. These measurements were performed by a single examiner (R.K.) with 5 years of experience in shear wave elastography measurement. For each image, a region of interest (ROI) in the color map of shear wave elastography was set as large as possible while excluding artifacts (saturated area), missing areas (unfilled region within the elasticity map), subcutaneous fat, fascia, and aponeurosis. The Young's modulus over ROI was spatially averaged, and the obtained Young's modulus was divided by 3 to calculate the muscle shear modulus [23]. Five and two measured values of VL shear modulus were averaged at 0° of knee flexion and the other knee joint angles for further analysis, respectively.

**FIGURE 1:**
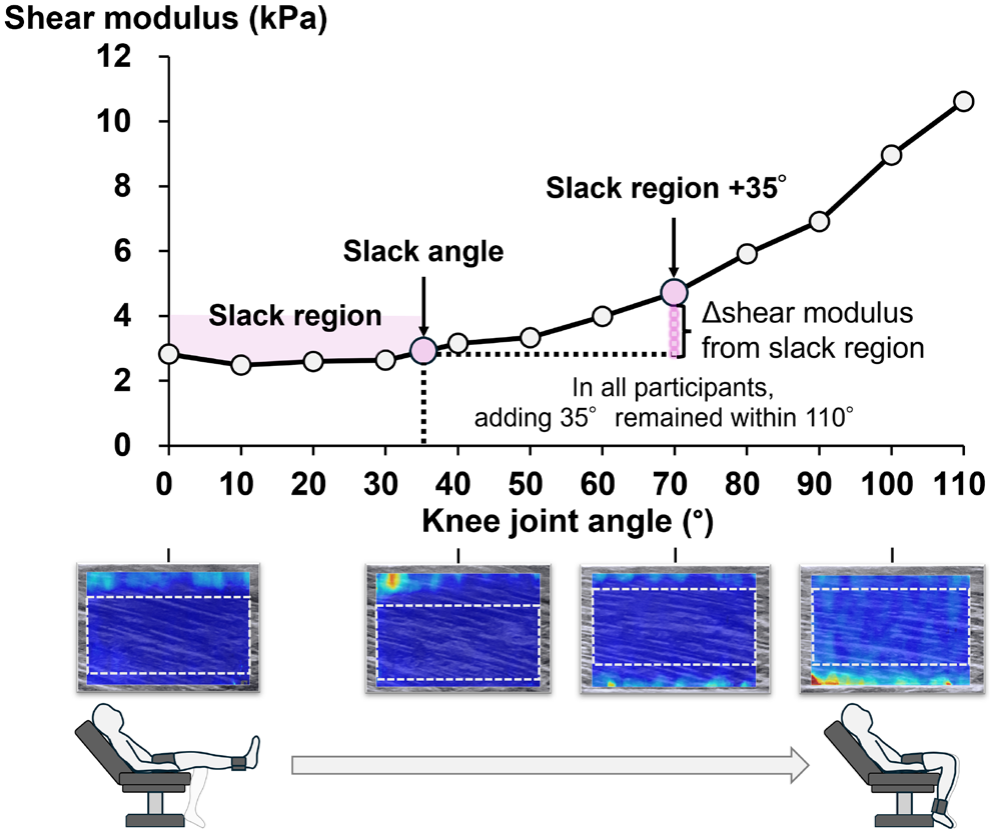
Representative images of shear modulus for the vastus lateralis and definitions of shear modulus variables.

The shear modulus of VL at each knee joint angle was used to calculate muscle slack angle (the onset of the rise in passive tension) for each participant based on the following procedures. The VL is theoretically slack at the initial position of shear modulus measurement (0° of knee flexion), and thus the slack angle could be identified as the angle at which the shear modulus first increased during passive knee flexion from the initial joint angle based on previous procedures [24, 25]. The onset was defined as the shear modulus value that first exceeded 2 standard deviations of that in the initial joint angle [25]. As the onset based on this definition is highly sensitive to variability in the shear modulus at the initial joint angle, the mean of five shear modulus values was used at 0° of knee flexion. Additionally, since shear modulus was measured at 10° increments to minimize measurement time in the present study, the actual onset of increase may have occurred between two consecutive measurement angles. For example, if the onset defined above was observed at 30° of knee flexion, the actual increase might have started between 20° and 30°. To account for this limitation, the slack angle was defined as the midpoint between the identified onset angle and the preceding angle (e.g., 25° when onset was detected at 30°). Moreover, an experienced examiner visually inspected the shear modulus–angle relationship plotted for each participant and confirmed that the identified slack angle corresponded to the point of increasing shear modulus for all participants. Shear modulus from 0° of knee flexion to the slack angle (mean value in adjacent two joint angles) was averaged and defined as shear modulus at slack region where the muscle is not lengthened. Additionally, the Δshear modulus from the slack region to slack angle +35° was also calculated as a region where the muscle is lengthened (Figure 1). The angle of slack region +35° was selected because it could be reached by all participants. In other words, as the maximal slack angle observed across participants was 75°, adding 35° remained within 110° (i.e., the maximal knee flexion angle assessed in this study).

### 2.3 Electromyographic Activity

Electromyographic (EMG) activity of VL was assessed during the shear modulus measurement to ensure whether this measurement was performed at rest. Pairs of pre‐amplified electrodes (DL‐141, S&ME, Japan, inter‐electrode distance: 12 mm) were positioned at 55% of thigh length in VL to avoid contact with the ultrasound scanner transducer. A reference electrode was attached to the right patella. The EMG signals were recorded simultaneously with torque and joint angle signals at 1 kHz and then band‐pass filtered between 5 and 500 Hz using analysis software (LabChart ver. 8, ADInstruments, Australia). The root‐mean square value of the EMG data (RMS‐EMG) of VL was averaged across five or two shear modulus measurements at 0° of knee flexion and other knee flexion angles, respectively. In six participants, extremely high amplitudes in VL were observed, possibly due to apparent noise contamination. Thus, the EMG data from these participants were removed for further analysis (remaining *n* = 40). After shear modulus measurement, the participants performed five submaximal contractions at 30° of knee and hip flexion with the arms crossed over the chest, followed by two maximal voluntary contractions (3 s) of isometric knee extension. A rest period of 2 min was allowed between trials. For the trial with higher torque, the RMS‐EMG during maximal voluntary contraction of knee extension was calculated over a 0.3 s period around the peak torque. The RMS‐EMG during shear modulus measurements was normalized to that during maximal voluntary contraction at each knee joint angle as %RMS‐EMG.

### 2.4 Carnosine Concentration

**FIGURE 2:**
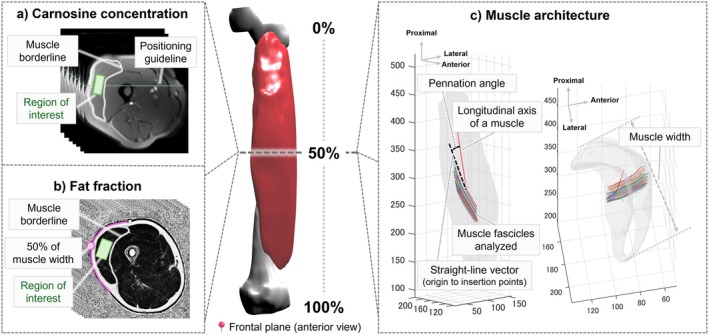
Measurement region of compositional and architectural variables.

The participants lay in a supine position with their hips and knees fully extended in the magnet bore of a 3‐T MR scanner (MAGNETOM Skyra, Siemens Healthineers, Germany). The right thigh was secured with a flexible wrap coil. Muscle carnosine content of ROI was quantified once using ^1^H‐MRS, based on the size of the carnosine peak at 8 ppm [26]. Muscle carnosine content has been reported to be positively associated with the percentage area of type II muscle fibers [12] and has thus increasingly been used as a non‐invasive proxy of muscle fiber typology in recent studies [12, 27]. A single‐voxel point‐resolved spectroscopy (PRESS) sequence was used for the carnosine scan with the following parameters: repetition time of 4500 ms, echo time of 33 ms, 64 excitations, 1024 data points, spectral bandwidth of 2000 Hz, acquisition time of 4.48 min, and a voxel size of 40 × 12 × 30 mm. The voxel was centered at 50% of thigh length in the sagittal plane and at the midline of VL in the coronal plane, with careful positioning confirmed across all imaging planes to exclude subcutaneous fat and adjacent muscle tissue (Figure 2a). These procedures were applied to all participants. After automatic and manual shimming procedures, the mean full width at half maximum (FWHM) was 24.9 ± 2.1 Hz.

The carnosine content (mM) was calculated using unsuppressed water (eight excitations) as an internal reference [28]. The formula used for this internal reference technique is as follows:

$$\begin{array}{c} \text{Carnosine concentration}\;\left(\mathrm{mM}\right)=\left[\left({\text{Signal}}_{\text{carnosine}}/{\text{Signal}}_{\text{water}}\right)\right]\\ \;\times \left[\left(\text{Correction factor}\;T1_{\text{water}}\right)/\left(\text{Correction factor}\;T1_{\text{carnosine}}\right)\right]\\ \;\times \left[\left(\text{Correction factor}\;T2_{\text{water}}\right)/\left(\text{Correction factor}\;T2_{\text{carnosine}}\right)\right]\\ \;\times \left(\text{Concentration of water in the muscle}\right)\times \text{number of protons in water} \end{array}$$

The relaxation correction factors for carnosine were described earlier by a previous study [28] and the correction factors for water by another study [29]. The concentration of water in a muscle was deduced from the molar concentration of water (55 000 mM) and the approximate water content of skeletal muscle tissue (0.7 L/kg wet weight of tissue [30]). These parameters were included in the following equation.

$$\begin{array}{c} \text{Carnosine concentration}\;\left(\mathrm{mM}\right)=\left[\left({\text{Signal}}_{\text{carnosine}}/{\text{Signal}}_{\text{water}}\right)\right]\\ \;\times \left[\left(1-\exp\left(-4500/1337.6\right)\right)/\left(1-\exp\left(-4500/1718\right)\right)\right]\\ \;\times \left[\left(\exp\left(-33/274\right)\right)/\left(\exp\left(-33/123\right)\right)\right]\times \left(0.7\times 55000\right)\times 2 \end{array}$$

To ensure the spectral quality of the measurements, three criteria were evaluated: FWHM, linewidth, and standard deviation of signal amplitude. Spectral quality was considered acceptable if FWHM was ≤ 30 Hz [16] and the linewidth was ≤ 12.78 Hz [31]. Regarding standard deviation of signal amplitude, spectra were required to fall within the range of the mean ± two standard deviations of all participants. Data were included in the final analysis only if at least two of these three criteria were met. In the present study, data from two participants failed to meet these criteria and were consequently excluded from further analysis (remaining *n* = 44).

### 2.5 Fat Fraction & Muscle Architecture

The participants were positioned in a supine position with the hip flexed at 30° and the knee at the slack angle determined through shear modulus measurement in the magnet bore of MR scanner as the slack angle was achievable for all participants within the scanner. These joint angles were carefully adjusted by using a manual goniometer. To minimize contact between the coil and right VL, some cushions were placed on the shanks, left thigh, and upper body. The feet were placed into a handmade pad, and the lower legs (ankles and shanks) were secured with a non‐elastic belt to avoid hip and knee joint motions. To reduce the influence of fluid shifts on lower limb muscle morphology due to postural changes, participants lay supine for at least 20 min before scanning. Throughout the entire scanning protocols, participants were instructed to keep their legs motionless and to remain fully relaxed.

A series of axial MR images of the right thigh were acquired using a 32‐channel spine array coil and two 18‐channel body array coils (Body 18 and CP Spine Array Coil, Siemens Healthineers, Germany). Two sequences were used to cover the same field of view of the thigh: T1 VIBE two‐point DIXON (gradient recalled echo, echo times: 2.46 ms, repetition time: 5.98 ms, slice interval: 5 mm, field of view: 314 × 419 mm, reconstructed matrix size: 288 × 512, bandwidth: 610 Hz, flip angle: 12°, scan time: 19 s for a single scan) and DTI (spin echo‐echo planar imaging, echo time: 63 ms, repetition time: 3500 ms, slice interval: 5 mm, field of view: 315 × 420 mm, reconstructed matrix size: 90 × 120, number of averages: 1, *b* = 0 and 500 s/mm^2^, 12 gradient directions on a hemisphere, bandwidth: 3206 Hz, flip angle: 90°, scan time: 120 s for a single scan). In the axial plane images, the central slice was positioned at 50% of the thigh length to cover the region corresponding to the shear modulus measurement.

Based on water‐ and fat‐only images of DIXON sequence, a fat fraction map was calculated using the ratio of water and fat signal intensities within each voxel of the images.

$$\mathrm{fat}\;\text{fraction}=\frac{I_{\mathrm{fat}}}{I_{\mathrm{fat}}+I_{\text{water}}}\times 100$$

where *I*_fat_ is the signal intensity from the fat‐saturated image and *I*_water_ is the signal intensity from the water‐saturated image. For each of the seven consecutive slices (corresponding to a longitudinal ultrasound transducer width of approximately 3 cm) centered on the region of shear modulus measurement, 50% of VL width was identified using Image J software (1.54, National Institutes of Health [Figure 2b]). The ROI was then set at this position to ensure spatial correspondence with the ROI used for the shear modulus measurement. In each slice, the intramuscular fat fraction of VL was calculated twice as the average value of all voxels within the ROI. The two values were then averaged for each slice, and the mean value across all seven slices was used for subsequent analysis.

The analyzes of muscle architecture were conducted based on previously established procedures [8, 9]. In the in‐phase images of the DIXON sequence, the muscle boundary of VL was semiautomatically segmented from its origin to insertion. This segmentation was performed using a deep learning model based on the nn‐U‐Net framework [32], implemented within 3D Slicer. The model was specifically trained for the present study using manually segmented VL data from 13 individuals who were recruited for the present project. When errors and overlaps between adjacent muscle cross‐sections were identified, the outline of VL was manually traced. The diffusion‐weighted images acquired in DTI were denoised using local principal component analysis. The denoised images were then resampled by 3D interpolation to match the DIXON matrix size and voxel dimensions, and imported into DSI Studio to compute the primary eigenvector and fractional anisotropy for each voxel. Fiber tracking was initiated from a seed point randomly placed within VL of the right thigh and propagated bidirectionally with a step size of 0.1 mm. Tracking was terminated when fractional anisotropy fell below 0.1 or when the turning angle between successive steps exceeded 30°. In addition, tracked fiber tracts shorter than 20 mm or longer than 200 mm were discarded. These thresholds were determined during preliminary testing to enhance intramuscular fiber delineation while reducing anatomically implausible trajectories, with reference to previous anatomical study [33]. DTI‐derived muscle architecture measurement is reported to be relatively robust to reasonable variations in tracking settings [34]. Fiber tracking was repeated until 5000 fiber tracts were obtained for each muscle. The tracked fiber tracts represent the length and orientation of muscle fascicles.

The 3D coordinate data of the segmentation and tracked fiber tracts were exported to MATLAB R2024a (MathWorks, Natick, USA). Principal component analysis was applied to the point cloud representing VL. The first principal component was defined as the longitudinal axis of the muscle, whereas the second principal component was defined as the short axis, corresponding to the apparent muscle width. To correspond with the measurement region of shear modulus, only fiber tracts whose midpoint was located within four slices anterior and posterior to the slice corresponding to 50% of thigh length were included in the analysis (Figure 2c). Additionally, the fiber tract midpoint was required to be positioned between 45% and 55% of the muscle width, defined as the linear distance between the most medial and lateral points along the short‐axis direction. Based on previous definition [35], the pennation angle was defined as the angle (°) between the fascicle direction vector (the straight‐line vector connecting the origin and insertion points of the tracked fiber tract) and the muscle longitudinal axis vector (first principal component derived from principal component analysis of the segmented muscle belly). In this study, the 3D pennation angle was calculated to account for sagittal and coronal deviations. Fascicle length was defined as the sum of distances between consecutive points along the fiber tracts. For the fascicle length and pennation angle, the median value among the tracked fascicles was used to minimize the possible effects of outlier values on the measurement data. Previous DTI studies established the validity that tracked fiber architecture represents muscle fascicle architecture [8, 36].

### 2.6 Knee Extensor MA

In the same position as fat fraction and muscle architecture measurements, T1‐weighted MR images of the right knee joint were obtained in the sagittal, coronal, and axial planes using a 32‐channel spine array coil and 18‐channel body array coils with the following parameters: field of view, 260 × 260 mm; matrix, 512 × 512; slice thickness, 5 mm; pixel size, 0.51 × 0.51 mm; repetition time, 472 ms; echo time, 14 ms; gap, 5 mm; the number of slices, 18 per block. The central slice in the axial plane images was positioned at 100% of the thigh length (the popliteal crease) to encompass the knee joint region.

**FIGURE 3:**
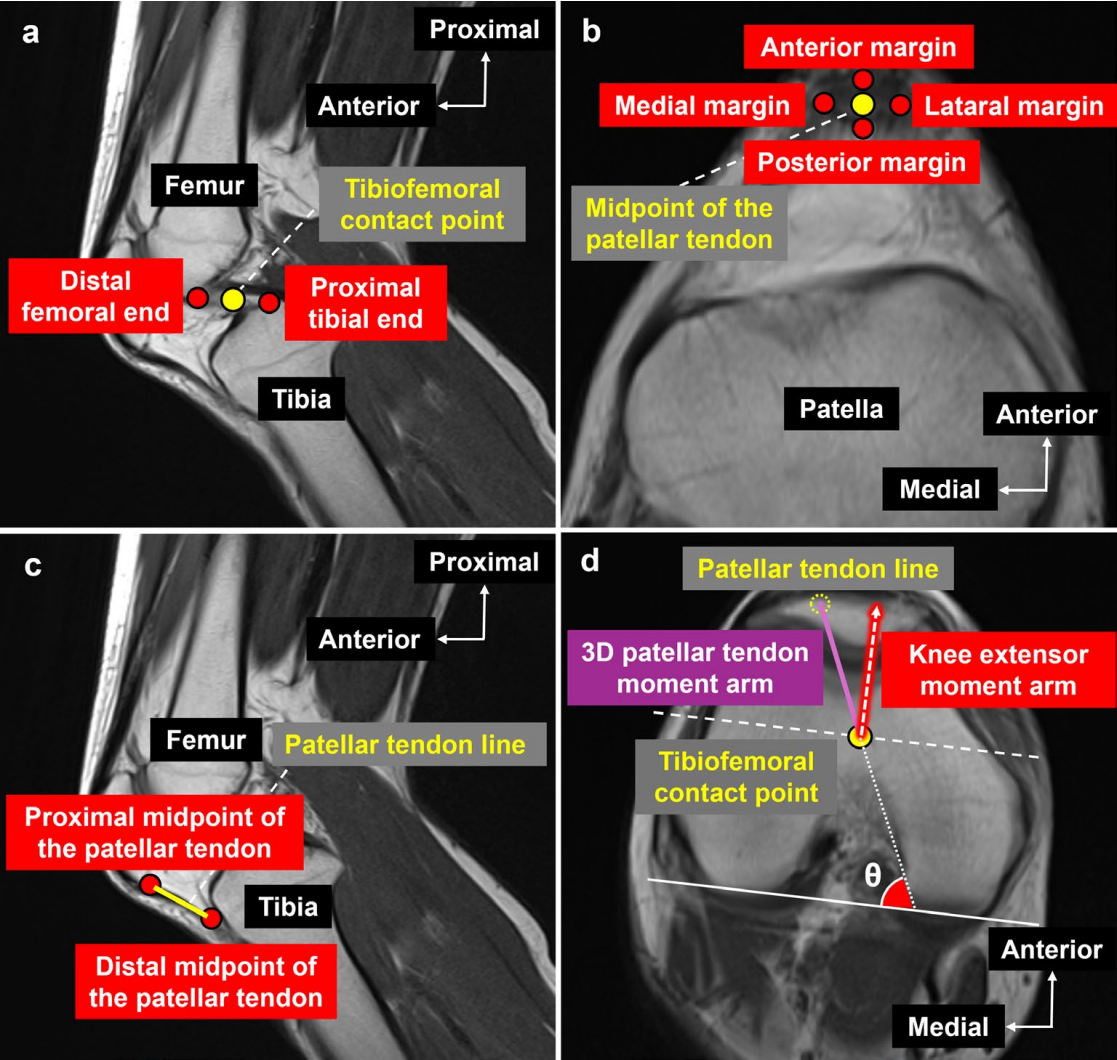
Determination of knee extensor moment arm. Step 1: (a) The tibiofemoral contact point (yellow dot) was identified in the sagittal plane as the midpoint between the distal femoral end and the proximal tibial end (red dots) on the center of the femoral trochlear groove. Step 2: (b) The intersection of the anterior–posterior and medial‐lateral margins (red dots) of the patellar tendon was identified on the transverse image to determine the midpoint of the patellar tendon (yellow dot). Step 3: (c) The midpoints at both the proximal and distal regions of the tendon (red dots) were then identified, and the line connecting these points was defined as the patellar tendon line (yellow straight line). Step 4: (d) A perpendicular line was drawn from the contact point of the tibiofemoral joint to the patellar tendon line which was defined as 3D‐MA (purple straight line). The angle (θ) on the transverse plane was calculated between the tangent to medial‐lateral posterior condyle of the femur and the 3D‐MA. Finally, the angle (θ) was used to decompose the 3D‐MA into individual plane components, and the length (cm) of the sagittal plane component was defined as knee extensor MA (white and red broken line).

The patellar tendon MA was assessed based on the procedures of a previous study (Figure 3) [37]. The following landmarks were identified for measuring the patellar tendon MA using 3D Slicer, and spatial coordinates were obtained using a multiplanar reconstruction function. The patellar tendon MA was defined as the distance between the contact point of the tibiofemoral joint and the patellar tendon line (described later) [37]. The tibiofemoral contact point was identified in the sagittal plane as the midpoint between the distal femoral end and the proximal tibial end on the center of the femoral trochlear groove (Figure 3a). To determine the midpoint of the patellar tendon, the intersection of the anterior–posterior and medial‐lateral margins of the patellar tendon was identified on the transverse image (Figure 3b). The midpoints at both the proximal and distal regions of the tendon were then identified, and the line connecting these points was defined as the patellar tendon line (Figure 3c). Using the 3D spatial coordinates, a perpendicular line was drawn from the contact point of the tibiofemoral joint to the patellar tendon line which was defined as 3D‐MA. The angle (θ) on the transverse plane was subsequently calculated between the tangent to the medial‐lateral posterior condyle of the femur and the 3D‐MA (Figure 3d). Finally, the angle (θ) was used to decompose the 3D‐MA into individual plane components, and the length (cm) of the sagittal plane component was defined as knee extensor MA. The knee extensor MA was calculated twice, and the mean value of two trials was used for further analysis. The fascicle length was divided by the knee extensor MA to determine the fascicle length to MA ratio, as the change in muscle‐tendon unit length for a given joint angle change depends on MA.

### 2.7 Intra‐Day Measurement Reliability

Six healthy male participants were recruited to test the intra‐day measurement reliability of shear modulus from 0° to 110° of knee flexion, carnosine concentration, fat fraction, fascicle length, 3D pennation angle, and knee extensor MA. All variables were measured twice on the same day, with an interval of 1 to 20 min between measurements. For the shear modulus at each knee joint angle, fat fraction, and knee extensor MA, each measurement was analyzed twice, and the mean of the two analyzes was used for subsequent analyzes. The carnosine concentration, fascicle length, and 3D pennation angle were analyzed once per measurement. Intra‐day measurement reliability was evaluated using intraclass correlation coefficients (ICC_[1,2]_ or ICC_[1,1]_, as appropriate) and CV.

### 2.8 Statistics

The Shapiro–Wilk test was performed to assess data normality of primary variables (shear modulus at slack region and Δshear modulus in the lengthened position, carnosine concentration, fat fraction, fascicle length to MA ratio, and pennation angle). Two variables (Δshear modulus in the lengthened position and fascicle length to MA ratio) were shown to be non‐Gaussian (*p* < 0.016), and these data were thus log10 transformed for further analysis. Pearson's product moment correlation coefficients and their 95% confidence intervals (CIs) were calculated to examine the relationship between shear modulus variables (shear modulus in the slack region and Δshear modulus in the lengthened position) and slack angle. Additionally, these statistical variables were calculated to examine the relationship between shear modulus variables and other primary measurement variables. Correlation coefficients were categorized as “weak” (*r* < 0.40), “moderate” (*r* = 0.40–0.60), “strong” (*r* = 0.61–0.79), or “very strong” (*r* = 0.80–1.00) [38]. Considering multiple tests, the *p* value was corrected based on the number of dependent variables using the Benjamini–Hochberg method [39] at a false discovery rate of < 0.05. When two or more significant correlations were observed for an independent variable, a stepwise multiple linear regression analysis was performed to quantify the variance in the dependent variable explained by the independent variables. The statistical power was calculated for these tests. The CV for individual variability in each primary measurement variable was also calculated. The significance level was set at *α* = 0.05. Data are presented as mean ± standard deviation. All statistical analyzes were performed using statistical software packages (IBM SPSS Statistics, version 28.0, IBM Corporation, Armonk, USA and R software, version 4.4.1, R Foundation for Statistical Computing, Vienna, Austria).

## 3 Results

The results of the intra‐day measurement reliability for the primary variables are presented in Table S1 of the Supporting Information. The ICCs ranged from 0.80 to 0.99, and the CVs ranged from 2.2% to 6.5% across all primary variables measured.

**FIGURE 4:**
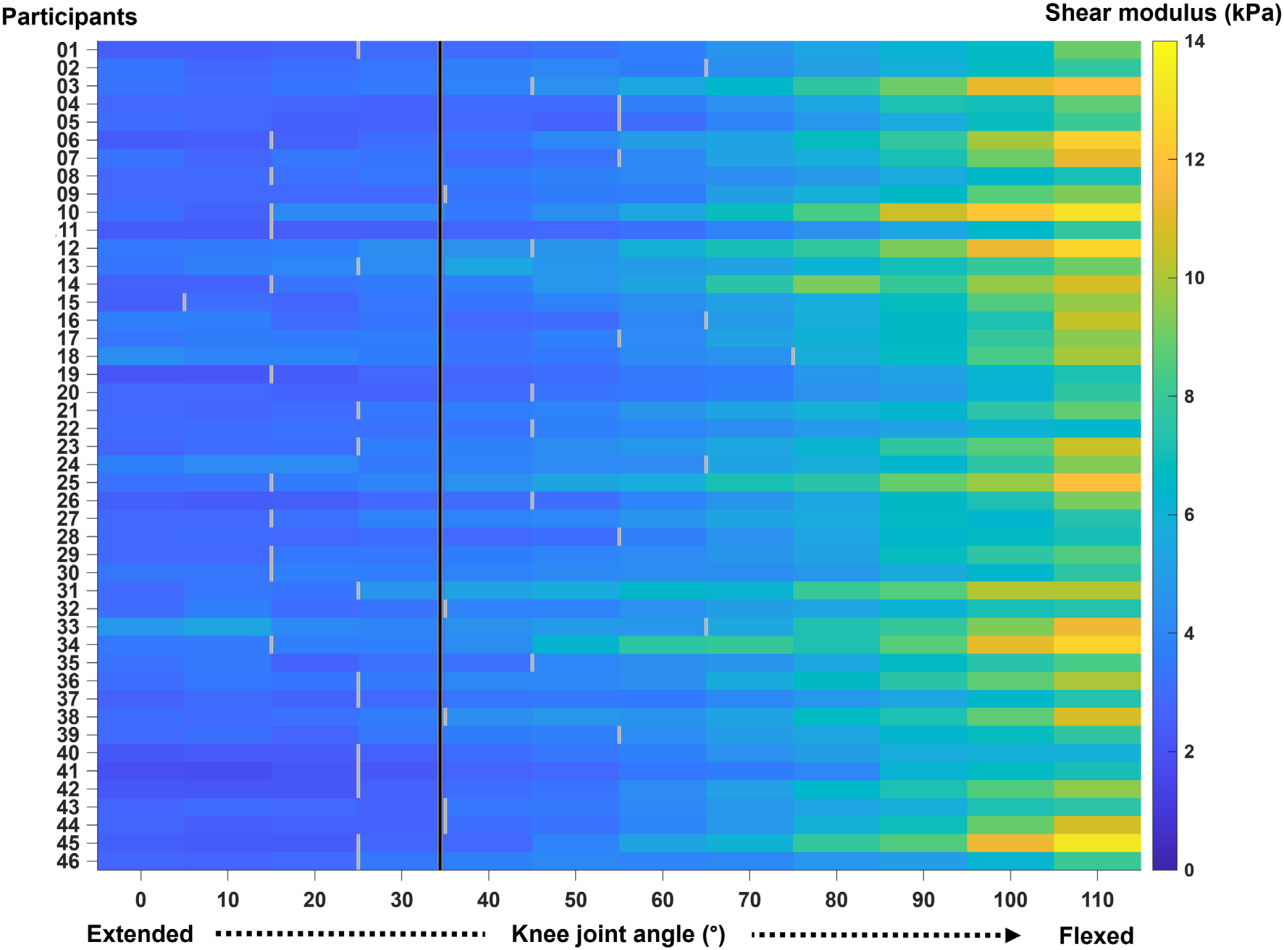
Individual data on shear modulus of the vastus lateralis from 0° to 110° of knee flexion. The black vertical line indicates the mean slack angle across all participants, while the gray vertical lines represent the individual slack angles for each participant.

The data for the primary variables are shown in Table 1. All individual data on VL shear modulus from 0° to 110° of knee flexion are represented in Figure 4. The shear modulus in the slack region ranged from 2.1 to 4.7 kPa, whereas the Δshear modulus in the lengthened position ranged from 0.5 to 4.8 kPa. Consistent with this observation, the CV for Δshear modulus in the lengthened position (54.5%) was more than threefold greater than that for the shear modulus in the slack region (16.4%). The %RMS‐EMG of VL during the shear modulus measurements was 0.4% ± 1.0% (0.1%–4.8%) throughout all measurement angles across all participants.

**TABLE 1:** Data for the primary variables.

Outcomes	Values	Coefficient of variation (%)
Age (yr)	22.8 ± 4.2	
Body mass (kg)	64.3 ± 9.0	
Height (cm)	172.2 ± 4.8	
Shear modulus at 0° (kPa)	3.00 ± 0.52	17.4
Shear modulus at 10° (kPa)	3.06 ± 0.60	19.6
Shear modulus at 20° (kPa)	3.10 ± 0.53	17.0
Shear modulus at 30° (kPa)	3.33 ± 0.52	15.6
Shear modulus at 40° (kPa)	3.56 ± 0.62	17.3
Shear modulus at 50° (kPa)	3.90 ± 0.73	18.8
Shear modulus at 60° (kPa)	4.39 ± 0.87	19.9
Shear modulus at 70° (kPa)	5.08 ± 1.01	19.9
Shear modulus at 80° (kPa)	5.94 ± 1.17	19.6
Shear modulus at 90° (kPa)	6.85 ± 1.24	18.2
Shear modulus at 100° (kPa)	8.02 ± 1.65	20.5
Shear modulus at 110° (kPa)	9.29 ± 1.90	20.4
Slack angle (°)	35.00 ± 17.89	51.1
Shear modulus at slack region (kPa)	3.11 ± 0.51	16.4
Δshear modulus at the +35° from the slack region (kPa)	2.18 ± 1.19	54.5
Carnosine concentration (mM)	6.67 ± 1.60	24.0
Fat fraction (%)	7.61 ± 1.69	22.2
Fascicle length (mm)	50.61 ± 7.33	14.5
3D pennation angle (°)	34.09 ± 6.15	18.0
Knee extensor moment arm (mm)	29.78 ± 3.22	10.8
Fascicle length to moment arm ratio (a.u.)	1.73 ± 0.37	21.6

*Note:* Values indicate mean ± standard deviations across the participants (*n* = 44 or 46). Coefficient of variation represents inter‐individual variability in each measurement variable.

**FIGURE 5:**
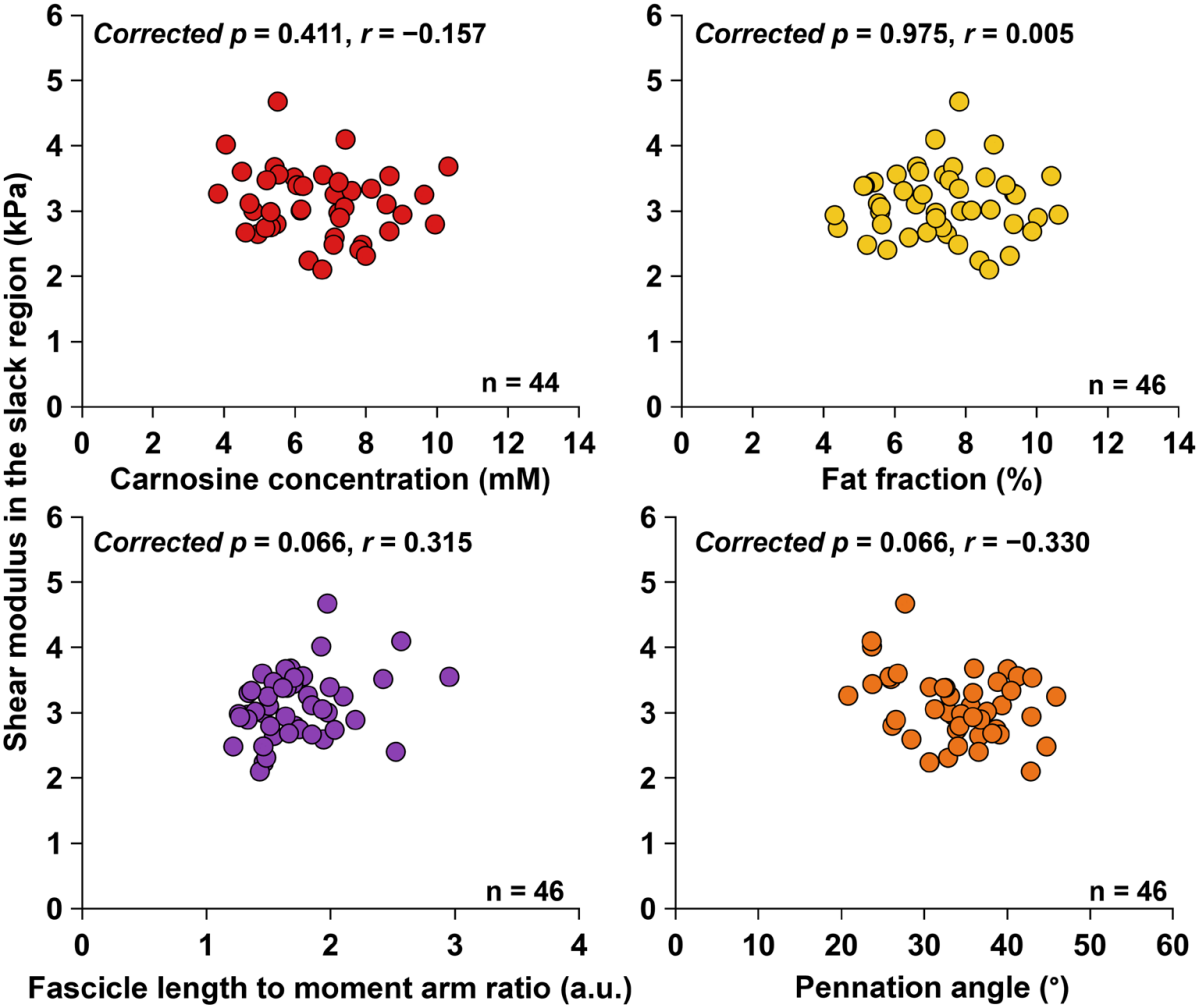
Association between the shear modulus in the slack region and measurement variables in the vastus lateralis.

**FIGURE 6:**
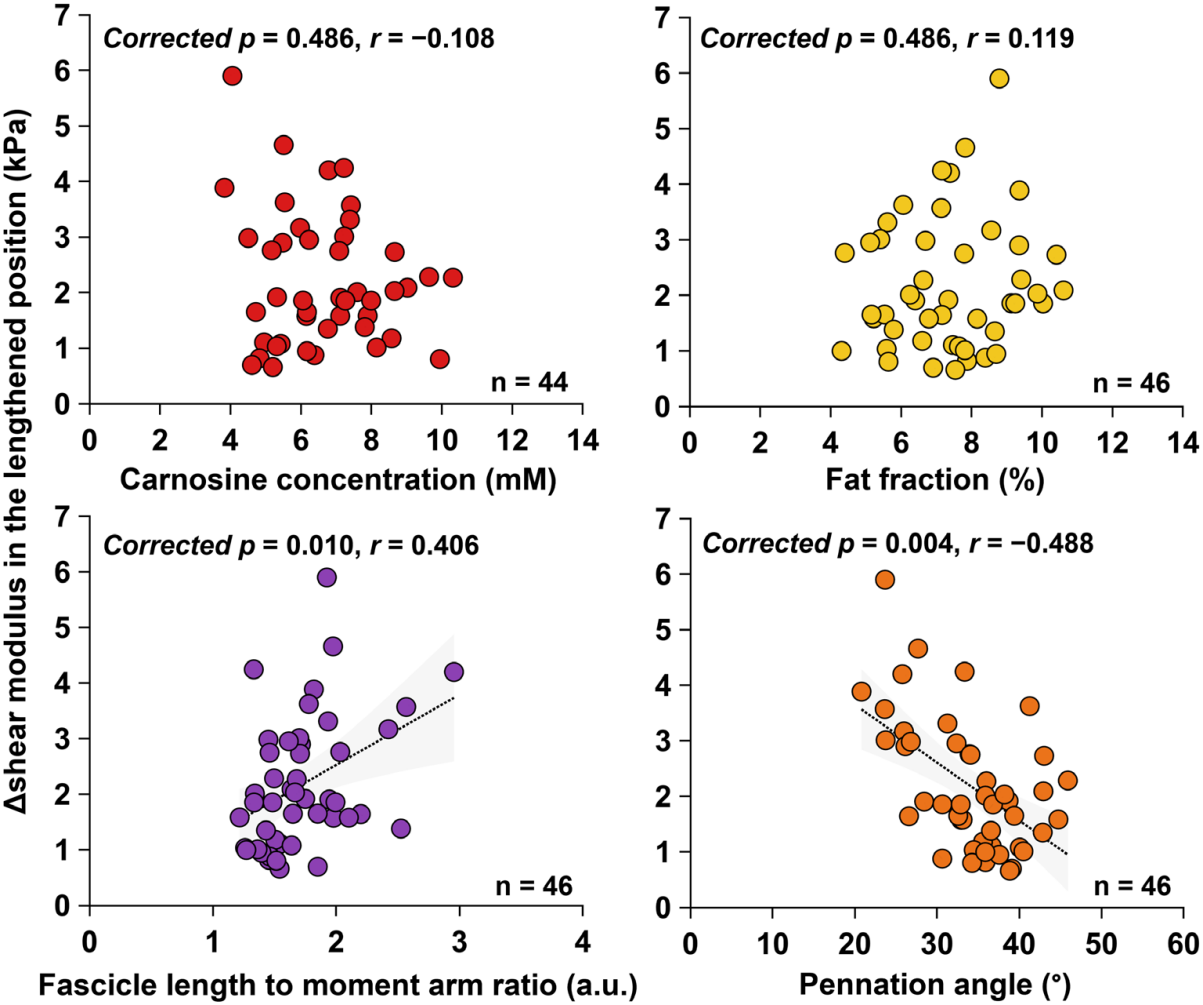
Association between the Δshear modulus of the vastus lateralis in the lengthened position (+35° from the slack region) and measurement variables in the vastus lateralis. The dotted line indicates the linear regression fit. The shaded area denotes the 95% confidence interval for the fitted mean.

The slack angle was significantly and positively correlated with VL shear modulus in the slack region (corrected *p* = 0.002, *r* = 0.446, 95% CI: 0.179–0.652, statistical power = 0.888) and Δshear modulus in the lengthened position (corrected *p* < 0.001, *r* = 0.766, 95% CI: 0.612–0.864, statistical power = 0.999). There were no significant correlations between VL shear modulus at slack region and primary variables (corrected *p* = 0.066–0.975, *r* = −0.330 to 0.315, 95% CI: −0.566 to 0.555, statistical power = 0.050–0.580 [Figure 5]). The Δshear modulus in the lengthened position showed a significant positive correlation with the fascicle length to MA ratio (corrected *p* = 0.010, *r* = 0.406, 95% CI: 0.131–0.623, statistical power = 0.816) and a significant negative correlation with pennation angle (corrected *p* = 0.004, *r* = −0.488, 95% CI: −0.682 to −0.230, statistical power = 0.942), but not with carnosine concentration (corrected *p* = 0.486, *r* = −0.108, 95% CI: −0.392 to 0.195, statistical powe*r* = 0.107) or fat fraction (corrected *p* = 0.486, *r* = 0.119, 95% CI: −0.177 to 0.396, statistical power = 0.124 [Figure 6]). Stepwise multiple regression analysis identified pennation angle as the only significant predictor of the Δshear modulus in the lengthened position, explaining approximately 20% of the variance (*R* ^2^ = 0.238, adjusted *R* ^2^ = 0.221, *F*(1,44) = 13.749, *p* < 0.001, statistical power = 0.916).

## 4 Discussion

We found that the inter‐individual variability was substantially greater for the Δshear modulus in the lengthened position (54.5% [CV]) than for the shear modulus in the slack region (16.4%). The shear modulus in the slack region was not associated with any of the measured variables, whereas the Δshear modulus in the lengthened position was significantly and negatively associated with pennation angle, accounting for only ~20% of the variance. Although substantial inter‐individual variability in passive muscle mechanical properties has been observed in several studies [1, 2, 22], the contribution of compositional and architectural factors to this variability has remained largely unclear, except for intramuscular fat content [11]. Hence, the present study provides the first in vivo evidence suggesting that (1) the determinants of passive muscle shear modulus are dependent on muscle length, (2) pennation angle is associated when the muscle is lengthened for a given change in joint angle, and (3) pennation angle alone explains only a limited part of the inter‐individual variability in passive shear modulus change. Overall, our results imply that the measured compositional and architectural factors are insufficient to fully account for the observed inter‐individual variability in VL passive shear modulus, highlighting the need to explore additional relevant factors underlying shear modulus variability.

In the present study, CV for Δshear modulus in the lengthened position (54.5%) was more than threefold greater than that for the shear modulus in the slack region (16.4%). These results highlight that the magnitude of inter‐individual variability in passive muscle shear modulus is muscle length‐dependent; specifically, such variability may be limited at shortened muscle lengths and becomes markedly more pronounced at longer muscle lengths. Similar observations were also made in a previous study [17]. Additionally, the change in shear modulus in the lengthened position was strongly and positively correlated with the slack angle in the present study, suggesting that inter‐individual variability in change in passive muscle shear modulus for a given change in joint angle is largely determined by the slack angle. Meanwhile, in this study, the 3D pennation angle of VL assessed at the slack angle was negatively correlated with the Δshear modulus in the lengthened position, but not with the shear modulus at the slack region. Pennation angle was selected as a predictor of the Δshear modulus in the lengthened position, explaining approximately 20% of the variance. These findings indicate that individuals with a larger pennation angle experience a smaller increase in passive muscle shear modulus for a given change in joint angle, which partially supports our hypothesis. To date, pennation angle might have received relatively little attention as a potential determinant of passive muscle mechanical properties [40], but the present study is the first to show pennation angle as a factor that is associated with inter‐individual variability in changes in passive muscle shear modulus when the muscle is lengthened.

A negative correlation between the Δshear modulus in the lengthened position and pennation angle could be partly explained by rotation of fascicles (i.e., gearing effect). In pennate muscles, fascicles have been shown to rotate in both the coronal and sagittal planes during passive muscle lengthening [9]. Owing to this 3D fascicle rotation, muscle belly lengthening was shown to be approximately 38% greater than the corresponding fascicle elongation of the human medial gastrocnemius [9]. The gearing effect is advantageous in reducing fascicle elongation associated with a given muscle belly elongation (Figure S1 of the Supporting Information). Additionally, the initial pennation angle was shown to be positively correlated with the magnitude of the gearing effect of the muscle belly [9]. Considering these findings, it is plausible that individuals with larger pennation angles in the present study experienced greater fascicle rotation during a given change in joint angle, thereby attenuating fascicle elongation and, consequently, showing a smaller increase in passive muscle shear modulus. However, in this study, pennation angle was measured only at the slack angle, so we could not directly evaluate an association between the magnitude of actual fascicle rotation during a given joint angle change and the corresponding change in passive muscle shear modulus. Examination of this association remains an important topic for future research to further elucidate the underlying mechanism(s) of our findings.

The pennation angle of VL observed in the present study (34.09° ± 6.15°) appears to be larger than values reported in previous studies using cadaver‐based architecture datasets (e.g., 18.4° ± 6.8° [41]) and conventional 2D ultrasonography (14° to 18° [42]). Importantly, pennation angle was measured in different dimensions between the present and previous studies [41, 42]. Previous studies in VL typically measured 2D pennation angle within the sagittal plane only [41, 42], whereas the present study estimated 3D pennation angle by incorporating fascicle orientation in both the sagittal and coronal planes. A previous study reported that the discrepancy between 2D and 3D pennation angle estimates in bipennate muscles can be substantial, reaching up to 47.1% [35]. Hence, differences in measurement dimensionality likely account, at least in part, for the larger pennation angles obtained in the present study compared with previous studies [41, 42]. Moreover, as mentioned earlier, given that fascicles in pennate muscles rotate in both the sagittal and coronal planes during passive muscle lengthening [9], it is reasonable to adopt 3D pennation angle when examining the relationship between muscle architecture and passive shear modulus.

The fascicle length to MA ratio showed a positive correlation with the Δshear modulus in the lengthened position, but not with that in the slack region. However, this variable was not retained as a predictor in the multiple regression analysis for the Δshear modulus in the lengthened position, which is inconsistent with our hypothesis. A plausible explanation for this discrepancy between the simple correlation and multiple regression analyzes is the indirect influence of pennation angle. Because muscle architectural variables are interrelated, we further examined the association between the fascicle length to MA ratio and pennation angle using a Pearson's product‐moment correlation analysis. This analysis revealed a significant negative correlation between the two variables (*p* = 0.001, *r* = −0.482, 95% CI: −0.677 to −0.222), indicating that individuals with smaller pennation angles tended to have a larger fascicle length to MA ratio. Given that individuals with smaller pennation angles exhibited a greater increase in shear modulus in the lengthened position, the observed association between the fascicle length to MA ratio and the change in shear modulus is likely indirect and primarily driven by pennation angle itself. Taken together, these findings suggest that the fascicle length to MA ratio does not independently explain inter‐individual variability in changes in passive muscle shear modulus, at least within the range of muscle length changes examined in this study (+35° from the slack region). Because the magnitude of inter‐individual variability in fascicle length and moment arm was relatively smaller (14.5% and 10.8%, respectively) compared to 3D pennation angle (18.0%), these parameters may not exhibit sufficient variability to substantially influence passive muscle shear modulus.

Up to the slack region +35° adopted in this study, actual fascicle elongation may have been limited, as the overall muscle belly lengthening could be largely accompanied by architectural gearing effects. A previous study has reported that the pennation angle of VL gradually decreased over the entire range of the passive knee flexion [42]. Theoretically, a decrease in pennation angle would be expected to attenuate such gearing effects [9], potentially allowing the influence of the fascicle length to MA ratio on inter‐individual variability in shear modulus change to become more apparent at greater muscle lengths. To test this possibility, we examined the associations between changes in shear modulus and pennation angle as well as the fascicle length to MA ratio at each examined joint angle beyond the slack region using semi‐partial correlation analyzes, adjusting for the effects of fascicle length and MA as well as pennation angle, respectively. The semi‐partial correlation analysis revealed that pennation angle was significantly and negatively associated with Δshear modulus at +15°, +25°, +35°, and +55° from the slack region (corrected *p* = 0.015–0.020, *r* = −0.421 to −0.353 [Table S2 of the Supporting Information]). In contrast, the fascicle length to MA ratio showed no significant associations with changes in shear modulus at any of the examined joint angles beyond the slack region (corrected *p* = 0.290–0.861, *r* = −0.114 to 0.356 [Table S3 of the Supporting Information]). These findings allow us to speculate that the association of pennation angle to inter‐individual variability in passive muscle shear modulus diminishes depending on joint angle change when the muscle is substantially lengthened from the slack region, and that the fascicle length to MA ratio does not represent a robust determinant of such variability. However, the sample size gradually decreased with an increase in joint angle in the aforementioned analyzes. Thus, whether these observations would be replicated with a larger sample size with a consistent sample size across all joint angles remains an important topic for future investigation.

The present study showed that VL carnosine concentration was not associated with either the shear modulus in the slack region or the Δshear modulus in the lengthened position. These findings are inconsistent with previous evidence showing that slow‐twitch muscle fibers exhibit a higher Young's modulus (defined as elastic tension normalized to sarcomere strain) than fast‐twitch fibers [4]. In the previous study, muscle fiber bundles were isolated from rat soleus and extensor digitorum longus muscles, which predominantly consist of slow‐ and fast‐twitch fibers, respectively, and tensile testing was performed to separately estimate elastic, viscous, and viscoelastic components [4]. Meanwhile, the present study assessed VL shear modulus in vivo using ultrasound shear wave elastography. Importantly, the shear modulus measured by this technique reflects not only the mechanical properties of muscle fibers themselves but also those of other structural components within the muscle, such as collagenous connective tissues located between muscle fibers and fascicles. These non‐contractile tissues are known to substantially contribute to passive muscle mechanical properties, particularly during muscle elongation [24, 43] (discussed in the latter section), and may therefore mask the contribution of fiber‐type–related differences detectable at the fiber‐bundle level. Although muscle fiber composition has often been discussed as a possible determinant of inter‐population differences in passive muscle mechanical properties (e.g., lower passive muscle shear wave velocity in sprinters, who typically exhibit a greater proportion of fast‐twitch fibers, compared with endurance athletes, who are characterized by a higher proportion of slow‐twitch fibers) [44], the present findings suggest that, at least in human VL muscle assessed in vivo, the contribution of muscle fiber type composition per se to passive muscle shear modulus is likely limited.

In the present study, intramuscular fat fraction was not associated with either the shear modulus in the slack region or the Δshear modulus in the lengthened position. These findings are inconsistent with the previous study reporting a negative association between intramuscular fat content and change in shear wave velocity of the medial gastrocnemius from 0° to 15° of dorsiflexion in young healthy adults [11]. These discrepancies may be attributable, at least in part, to several methodological differences between the present study and a previous study. For example, the previous study [11] focused on the gastrocnemius, which has distinct architectural and compositional characteristics [45, 46, 47] with the VL examined in the present study. Also, the sample size was substantially smaller in the previous study (*n* = 20) than in the present study (*n* = 46), which may have limited statistical power. Additionally, intramuscular fat content was quantified using the DIXON method in the present study, whereas the previous study employed T1‐weighted imaging. The latter approach has been reported to overestimate intramuscular fat content, particularly in individuals with higher fat infiltration [47]. Although it is difficult to identify definitive reasons for the discrepancy between studies, the aforementioned factors may collectively be related to the inconsistent findings. Taken together, our findings suggest that intramuscular fat content is not a major contributor to inter‐individual variability in passive muscle shear modulus or its length‐dependent changes within VL.

### 4.1 Future Directions

The present findings indicate that the compositional and architectural factors assessed here are insufficient to fully account for individual variability in passive muscle shear modulus. This raises a fundamental question for future research: which additional factors contribute to the inter‐individual variability in passive muscle shear modulus in vivo? Although the determinants of inter‐individual variability in passive muscle shear modulus are likely multifactorial, some additional factors (both intra‐ and extra‐sarcomeric components) have been reported to influence passive muscle mechanical properties [20, 21, 22]. First, passive muscle mechanical properties were proposed to be influenced by the cycling dynamics of myosin–actin cross‐bridges, known as the main protein filaments of a sarcomere [20]. A previous study stated that, even in a relaxed state, a subset of cross‐bridges remains attached, thereby contributing to passive tension during sarcomere elongation. Second, passive muscle mechanical properties were also stated to be attributed to titin, a giant elastic protein that spans half of the sarcomere from the Z‐disc to the M‐line regions [21]. Titin functions as an intracellular spring, contributing to the centering of the myosin filament within the sarcomere and generating the passive tension in response to sarcomere elongation [21]. Finally, passive muscle mechanical properties were shown to be influenced by the intramuscular connective tissues, such as the endomysium and perimysium, which are rich in collagen with fibrous networks [5, 22]. During the early phase of muscle elongation, the collagen fibers are initially straightened and then elongated, thereby contributing to the development of passive tension during the late phase of muscle elongation [22]. To the best of our knowledge, limited evidence exists regarding how the aforementioned factors are directly related to inter‐individual variability in passive shear modulus of human skeletal muscle in vivo. The present study directs future research toward the investigation of more microscopic determinants beyond the compositional and architectural factors assessed here to better understand the mechanism(s) underlying inter‐individual variability in passive muscle shear modulus.

### 4.2 Limitations

This study has several limitations that should be considered. First, the onset of VL shear modulus was defined as the angle at which its shear modulus value first exceeded 2 standard deviations of that in the initial joint angle (i.e., 0° of knee flexion). The onset defined here may be influenced by the measurement errors of shear modulus for the initial joint angle. Considering this point, the mean of five shear modulus values was used in the initial joint angle. Additionally, we calculated ICC_(1,5)_ and CV for five shear modulus values in the initial joint angle. The results showed that the ICC_(1,5)_ and CV were 0.81 and 7.1% ± 3.8%, respectively. The ICC_(1,5)_ of the measured variables could be interpreted as at least “good” [48]. Thus, measurement variability at the initial joint angle is unlikely to have substantially influenced the determination of the onset of VL shear modulus in the present study. Second, Δshear modulus was calculated only at the +35° from the slack region, which represented the maximal range that all participants could reach within the measured knee flexion range (up to 110°). Hence, it remains unclear whether the findings obtained within this angular range can be extrapolated to joint angles beyond +35° from the slack region (e.g., into a potentially linear region). Future research should more comprehensively examine the associations between inter‐individual variability in shear modulus changes across various joint angles from the slack region and compositional and architectural variables of skeletal muscle. Additionally, as the main variables were assessed from a limited region (i.e., around 50% of thigh length) in this study, further studies are required to examine whether the factors related to inter‐individual variabilities in passive muscle shear modulus differ depending on the measurement region along the muscle. Moreover, the fascicle length was measured in this study; thus, we could not directly assess the number of sarcomeres in series. However, whether a muscle fascicle is long or short was reported to be determined more by the number of sarcomeres in series than by sarcomere length in a previous study using microendoscopy [49]. Hence, fascicle length may, at least in part, directly reflect the number of sarcomeres in series, although further research examining the relationship between serial sarcomere number and passive muscle shear modulus is warranted. Moreover, this study was limited to a specific population (young, healthy males) and a single pennate muscle (VL). Therefore, whether the present findings can be generalized to other populations or muscles (e.g., parallel‐fibered muscles) remains an important topic for future research. Furthermore, the present study assessed several compositional and architectural factors within the VL, which may interact with or counteract one another in influencing passive muscle mechanical properties. Thus, the results of the correlation analyzes should be interpreted as associations observed within this complex interplay among the measured variables. It should be noted that the absence of a significant bivariate association between shear modulus and a given factor does not necessarily indicate that the factor has little or no independent physiological contribution to passive muscle shear modulus. Additionally, the present study relied on Pearson correlation and linear regression analyzes to examine the associations between compositional and architectural factors and representative shear modulus variables. However, these relationships may not necessarily be linear, especially given the nonlinear nature of passive muscle mechanics and the complex biomechanical influence of architectural factors. To the best of our knowledge, there is currently limited evidence clearly demonstrating whether these relationships across individuals are linear or nonlinear. Thus, future studies using nonlinear analytical approaches (e.g., generalized additive models) may help to further clarify the potential mechanistic associations of those compositional and architectural factors with passive muscle mechanical properties. Finally, pennation angle induces an angular deviation between the muscle fascicle direction and the propagation direction of shear waves (i.e., the horizontal axis of the imaging plane), which may affect measured shear modulus values. However, a previous study directly examined this issue by comparing shear modulus measured with the probe parallel versus 20° oblique (a condition equivalent to the effect of pennation angle) to the fascicle direction within the same imaging plane and found that the influence was negligibly small (absolute difference: 0.5–0.6 kPa, relative difference < 1.3%), with ICC values of 0.979–0.998 [50]. Given that the pennation angles of VL in the present study ranged from 20.8° to 45.9° (i.e., a range of 25.1° across participants), which is close to the angular difference (0° vs. 20°) examined in the previous study [50], the potential bias induced by pennation angle on shear modulus measurements is likely minimal.

## 5 Conclusion

We found that the shear modulus in the slack region was not associated with any of the measured variables. Meanwhile, the Δshear modulus in the lengthened position was significantly negatively associated with pennation angle and positively associated with the fascicle length to MA ratio, while showing no association with carnosine concentration or intramuscular fat fraction. However, multiple regression analysis identified pennation angle as a single significant predictor of the Δshear modulus in the lengthened position, accounting for only ~20% of the variance. These findings suggest that (1) the determinants of passive muscle shear modulus are dependent on muscle length, (2) pennation angle is associated when the muscle is lengthened for a given change in joint angle, and (3) pennation angle alone explains only a limited part of the inter‐individual variability in passive shear modulus. Overall, our results imply that the measured compositional factors and architectural factors are insufficient to fully account for the observed individual variability in VL passive shear modulus at least within the angular range of this study, highlighting the need to explore additional relevant factors underlying shear modulus variability.

## 6 Perspective

Passive muscle shear modulus has been shown to exhibit adaptive plasticity in response to repeated mechanical loading, such as static stretching [51] and resistance training [23]. These adaptations are sometimes of particular interest for practitioners because of the physiological and clinical relevance. However, it remains unclear which factors are responsible for inducing chronic changes in passive muscle shear modulus. Our cross‐sectional study revealed a negative association of pennation angle with the Δshear modulus in the lengthened position. Some studies have reported that pennation angle substantially increases after resistance training including concentric contractions, whereas it remains largely unchanged after eccentric‐only training [14]. Thus, whether the change in pennation angle induced by the above training modality is associated with a corresponding change in passive muscle shear modulus represents an important topic for future longitudinal studies. The present cross‐sectional study serves as an important foundation for future longitudinal studies designed to elucidate factors associated with the plasticity of passive mechanical properties in skeletal muscle.

## Funding

This work was supported by JSPS KAKENHI (Grant 25K20979).

## Conflicts of Interest

The authors declare no conflicts of interest related to this study. The results of this study are presented clearly and honestly without fabrication, falsification, or inappropriate data manipulation. The results do not constitute an endorsement by the American College of Sports Medicine.

## Supporting information


**Figure S1:** Schematic illustrations explaining the potential mechanism underlying the observed association between the Δshear modulus of the vastus lateralis in the lengthened position and pennation angle in the sagittal plane. Individuals with larger pennation angles (left) may exhibit greater fascicle rotation during a given change in joint angle, thereby attenuating fascicle elongation and, consequently, showing a smaller increase in muscle shear modulus, whereas those with smaller pennation angles (right) may experience less fascicle rotation during a given joint angle change, leading to greater fascicle elongation and a larger increase in muscle shear modulus.

**Table S1:** Intra‐day measurement reliability of the primary variables (*n* = 6).

**Table S2:** Association between change in the shear modulus at each examined joint angle beyond the slack region and pennation angle in the vastus lateralis.
**Table S3:** Association between change in the shear modulus at each examined joint angle beyond the slack region and fascicle length to moment arm ratio in the vastus lateralis.

## Data Availability

The data that support the findings of this study are available from the corresponding author upon reasonable request.
