# Supplementary material for: Elucidation of Architectural and Compositional Factors Associated With Inter‐Individual Variability in Passive Shear Modulus of the Human Vastus Lateralis in Young Healthy Males
Source: Scand J Med Sci Sports. 2026 Jul 10;36(7):e70348. doi: 10.1111/sms.70348 (PMC13352097; doi:10.1111/sms.70348)
Supplement: Supplementary file 1 — Figure S1: Schematic illustrations explaining the potential mechanism underlying the observed association between the Δshear modulus of the vastus lateralis in the lengthened position and pennation angle in the sagittal plane. Individuals with larger pennation angles (left) may exhibit greater fascicle rotation during a given change in joint angle, thereby attenuating fascicle elongation and, consequently, showing a smaller increase in muscle shear modulus, whereas those with smaller pennation angles (right) may experience less fascicle rotation during a given joint angle change, leading to greater fascicle elongation and a larger increase in muscle shear modulus. [file SMS-36-e70348-s001.docx]

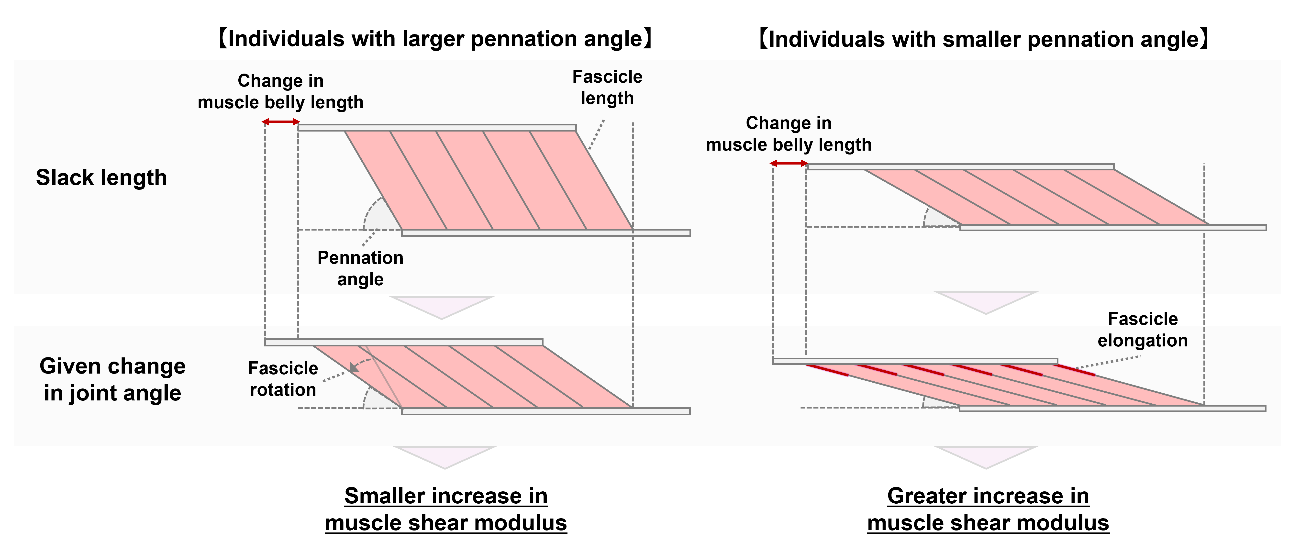
**Supplemental Fig 1. Schematic illustrations explaining the potential mechanism underlying the observed association between the Δshear modulus of the vastus lateralis in the lengthened position and pennation angle in the sagittal plane.** Individuals with larger pennation angles (left) may exhibit greater fascicle rotation during a given change in joint angle, thereby attenuating fascicle elongation and, consequently, showing a smaller increase in muscle shear modulus, whereas those with smaller pennation angles (right) may experience less fascicle rotation during a given joint angle change, leading to greater fascicle elongation and a larger increase in muscle shear modulus.
