# Supplementary material for: Elucidation of Architectural and Compositional Factors Associated With Inter‐Individual Variability in Passive Shear Modulus of the Human Vastus Lateralis in Young Healthy Males
Source: Scand J Med Sci Sports. 2026 Jul 10;36(7):e70348. doi: 10.1111/sms.70348 (PMC13352097; doi:10.1111/sms.70348)
Supplement: Supplementary file 2 — Table S1: Intra‐day measurement reliability of the primary variables (n = 6). [file SMS-36-e70348-s002.docx]

**Supplemental Table 1. Intra-day measurement reliability of the primary variables (n = 6).**

| Outcomes | Test | | |  | Re-test | | |  | ICC | CV (%) |
| --- | --- | --- | --- | --- | --- | --- | --- | --- | --- | --- |
| Shear modulus at 0° (kPa) | 2.90 | ± | 0.37 |  | 2.78 | ± | 0.22 |  | 0.85 | 4.5 |
| Shear modulus at 10° (kPa) | 2.70 | ± | 0.20 |  | 2.80 | ± | 0.22 |  | 0.95 | 3.2 |
| Shear modulus at 20° (kPa) | 3.09 | ± | 0.40 |  | 3.19 | ± | 0.55 |  | 0.95 | 3.8 |
| Shear modulus at 30° (kPa) | 3.29 | ± | 0.44 |  | 3.25 | ± | 0.36 |  | 0.80 | 4.8 |
| Shear modulus at 40° (kPa) | 3.34 | ± | 0.37 |  | 3.26 | ± | 0.18 |  | 0.82 | 4.2 |
| Shear modulus at 50° (kPa) | 3.74 | ± | 0.41 |  | 3.78 | ± | 0.37 |  | 0.94 | 2.7 |
| Shear modulus at 60° (kPa) | 4.45 | ± | 0.84 |  | 4.28 | ± | 0.59 |  | 0.90 | 6.5 |
| Shear modulus at 70° (kPa) | 5.21 | ± | 0.97 |  | 5.29 | ± | 0.86 |  | 0.94 | 4.3 |
| Shear modulus at 80° (kPa) | 6.22 | ± | 1.24 |  | 6.16 | ± | 1.32 |  | 0.98 | 3.7 |
| Shear modulus at 90° (kPa) | 7.33 | ± | 1.75 |  | 7.40 | ± | 1.77 |  | 0.98 | 3.7 |
| Shear modulus at 100° (kPa) | 8.71 | ± | 2.21 |  | 8.82 | ± | 1.96 |  | 0.96 | 4.7 |
| Shear modulus at 110° (kPa) | 10.17 | ± | 2.10 |  | 10.46 | ± | 2.36 |  | 0.99 | 2.2 |
|  |  |  |  |  |  |  |  |  |  |  |
| Carnosine concentration (mM) | 6.17 | ± | 1.88 |  | 6.02 | ± | 2.05 |  | 0.96 | 3.5 |
| Fat fraction (%) | 6.49 | ± | 0.99 |  | 6.79 | ± | 1.34 |  | 0.90 | 5.0 |
| Fascicle length (cm) | 5.41 | ± | 0.96 |  | 5.32 | ± | 0.71 |  | 0.90 | 3.9 |
| 3D Pennation angle (°) | 35.36 | ± | 5.72 |  | 36.97 | ± | 4.15 |  | 0.85 | 5.6 |
| Knee extensor moment arm (mm) | 29.89 | ± | 4.01 |  | 29.25 | ± | 4.30 |  | 0.83 | 5.1 |

Values indicate mean ± standard deviation across the participants (n = 6). ICC, intraclass correlation coefficient; CV, coefficient of variation. ICC _(1, 1)_ was calculated for carnosine concentration, fascicle length, and pennation angle, whereas ICC _(1, 2)_ was calculated for the other variables.
