# Supplementary material for: Elucidation of Architectural and Compositional Factors Associated With Inter‐Individual Variability in Passive Shear Modulus of the Human Vastus Lateralis in Young Healthy Males
Source: Scand J Med Sci Sports. 2026 Jul 10;36(7):e70348. doi: 10.1111/sms.70348 (PMC13352097; doi:10.1111/sms.70348)
Supplement: Supplementary file 3 — Table S2: Association between change in the shear modulus at each examined joint angle beyond the slack region and pennation angle in the vastus lateralis. Table S3: Association between change in the shear modulus at each examined joint angle beyond the slack region and fascicle length to moment arm ratio in the vastus lateralis. [file SMS-36-e70348-s003.docx]

**Supplemental Table 2. Association between change in the shear modulus at each examined joint angle beyond the slack region and pennation angle in the vastus lateralis.**

|  |  | **Pearson’s product moment  correlation analysis** | | | **Semi-partial**  **correlation analysis** | | |
| --- | --- | --- | --- | --- | --- | --- | --- |
| **Joint angle**  **from the slack region** | **n** | ***r*** | ***p*** | **Corrected *p*** | ***r*** | ***p*** | **Corrected *p*** |
| **5** | **46** | -0.462 | 0.001 | **0.002*** | -0.260 | 0.055 | 0.110 |
| **15** | **46** | -0.578 | <0.001 | **<0.001*** | -0.421 | 0.002 | **0.015*** |
| **25** | **46** | -0.574 | <0.001 | **<0.001*** | -0.393 | 0.003 | **0.015*** |
| **35** | **46** | -0.488 | 0.001 | **0.002*** | -0.353 | 0.008 | **0.020*** |
| **45** | **45** | -0.396 | 0.007 | **0.012*** | -0.212 | 0.129 | 0.215 |
| **55** | **41** | -0.501 | 0.001 | **0.002*** | -0.412 | 0.006 | **0.020*** |
| **65** | **35** | -0.208 | 0.231 | 0.289 | -0.030 | 0.857 | 0.936 |
| **75** | **29** | -0.131 | 0.499 | 0.554 | -0.114 | 0.569 | 0.711 |
| **85** | **24** | -0.009 | 0.967 | 0.967 | -0.018 | 0.936 | 0.936 |
| **95** | **12** | 0.458 | 0.135 | 0.193 | 0.407 | 0.183 | 0.261 |
| **105** | **1** | - | - | - | - | - | - |

*indicates a significant correlation between both variables (*p* < 0.05). The *p* value was corrected based on the number of correlations in each analysis (10) using the Benjamini and Hochberg method (Glickman et al. 2014) at a false discovery rate of < 0.05. “-” indicates that the analysis was not performed due to insufficient sample size.

**Supplemental Table 3. Association between change in the shear modulus at each examined joint angle beyond the slack region and fascicle length to moment arm ratio in the vastus lateralis.**

|  |  | **Pearson’s product moment  correlation analysis** | | | **Semi-partial**  **correlation analysis** | | |
| --- | --- | --- | --- | --- | --- | --- | --- |
| **Joint angle**  **from the slack region** | **n** | ***r*** | ***p*** | **Corrected *p*** | ***r*** | ***p*** | **Corrected *p*** |
| **5** | **46** | 0.370 | 0.011 | **0.022*** | 0.151 | 0.263 | 0.495 |
| **15** | **46** | 0.377 | 0.010 | **0.022*** | 0.115 | 0.355 | 0.507 |
| **25** | **46** | 0.402 | 0.006 | **0.020*** | 0.139 | 0.264 | 0.495 |
| **35** | **46** | 0.402 | 0.006 | **0.020*** | 0.157 | 0.220 | 0.495 |
| **45** | **45** | 0.425 | 0.004 | **0.020*** | 0.265 | 0.058 | 0.290 |
| **55** | **41** | 0.315 | 0.045 | 0.064 | 0.146 | 0.297 | 0.495 |
| **65** | **35** | 0.403 | 0.016 | **0.027*** | 0.356 | 0.034 | 0.290 |
| **75** | **29** | 0.028 | 0.673 | 0.841 | 0.034 | 0.861 | 0.861 |
| **85** | **24** | -0.063 | 0.771 | 0.857 | -0.097 | 0.660 | 0.787 |
| **95** | **12** | -0.039 | 0.903 | 0.903 | -0.114 | 0.708 | 0.787 |
| **105** | **1** | - | - | - | - | - | - |

*indicates a significant correlation between both variables (*p* < 0.05). The *p* value was corrected based on the number of correlations in each analysis (10) using the Benjamini and Hochberg method (Glickman et al. 2014) at a false discovery rate of < 0.05. “-” indicates that the analysis was not performed due to insufficient sample size.
